# Updates on the Treatment of Richter’s Syndrome, Including Novel Combination Approaches

**DOI:** 10.3390/cancers17060943

**Published:** 2025-03-11

**Authors:** Tanim Jain, Benjamin Heyman

**Affiliations:** 1 Division of Hematology-Oncology, Department of Medicine, University of California San Diego, La Jolla, CA 92093, USA; t7jain@health.ucsd.edu; 2 Division of Regenerative Medicine, Department of Medicine, UC San Diego Moores Cancer Center, University of California San Diego, La Jolla, CA 92093, USA

**Keywords:** Richter’s syndrome, diffuse large B cell lymphoma, chronic lymphocytic leukemia

## Abstract

Richter’s syndrome (RS) or transformation of chronic lymphocytic leukemia (CLL) into a more aggressive lymphoma (e.g., diffuse large B cell lymphoma, DLBCL) is a distinct disease that portends an overall poor prognosis and remains a challenge for clinicians to identify and treat effectively. This review of the current literature focuses on the pathology, diagnosis, and management of Richter’s syndrome. Clonally related RS has been found to have a worse prognosis than unrelated disease and the genomic profile of DLBCL-RS differs from that of de novo DLBCL. The standard of care therapy for RS has historically been chemoimmunotherapy; consolidative stem cell transplants have a role in improving durability of disease response. Given generally poor response rates to chemotherapy, there have been recent investigations into combination treatments with immune checkpoint inhibitors and small molecule targeted therapies, which have had mixed results. Additional studies are evaluating the use of bispecific antibodies, chimeric antigen receptor T cell therapy, and antibody drug conjugates. RS remains difficult to manage; however, advancements in the understanding of the underlying pathology of transformation and continued investigations into new therapies demonstrate promise for the future.

## Simple Summary

Chronic lymphocytic leukemia (CLL) is an indolent disease that can transform into more aggressive forms of lymphoma, termed Richter’s syndrome or transformation. This disease can be difficult to differentiate from aggressive CLL in terms of diagnosis and has historically been challenging to treat due to poor responses to chemotherapy. However, there have been recent studies evaluating the use of immunotherapy and small molecule targeted therapies for Richter’s syndrome, as single agents and in combination regimens, which have demonstrated some promise. This review aims to summarize the current understanding of the pathology, diagnosis, and treatment options for diffuse large B cell lymphoma-: Richter’s syndrome.

## 1. Introduction

Richter’s syndrome (RS) or transformation represents the transformation of chronic lymphocytic leukemia (CLL) from an indolent disease to an aggressive lymphoma. The occurrence of this transformation was first described by Maurice Richter in 1928 as a reticular cell sarcoma occurring in a patient with known CLL [1]. CLL has been shown to transform to diffuse large B cell lymphoma (DLBCL) most commonly, accounting for ~90% of cases; 5–10% of cases involve transformation to Hodgkin’s lymphoma (HL), and lymphoblastic lymphoma, histiocytic sarcoma, or other uncommon lymphomas account for <1% of cases [2]. Overall, DLBCL-RS has been associated with a poor prognosis, with median overall survival (OS) of less than 1 year. The focus of this review is on DLBCL-RS, including pathology, diagnosis, and treatment of this disease.

## 2. Epidemiology

The true incidence of RS development in patients with CLL has been difficult to ascertain due to a lack of uniformity in diagnosis, though estimates have varied between 2% and 10%, with recent studies reporting rates of 2–5% (Table 1) [3,4,5]. The median time to diagnosis of RS after diagnosis of CLL has similarly been variably reported, ranging from approximately 2 to 5 years [3,6,7]. Risk factors that have been associated with the development of RS include high risk genetic abnormalities (e.g., TP53 aberrancy, NOTCH1 mutation, del(11q)), unmutated IgHV, advanced Rai stage at diagnosis, expression of ZAP70, and SNPs in CD38 and LRP4 [3,8,9,10,11]. Of note, nearly half of patients diagnosed with DLBCL-RS may not have previously received treatment for CLL [3].

There has been some evidence of treatments for CLL predisposing patients to the development of RS. Fisher et al. (2016) noted almost twice as many cases of RS develop in patients treated with fludarabine and cyclophosphamide (FC) as compared with those treated with FC plus rituximab (FCR) [17]. Studies of ibrutinib for relapsed CLL demonstrated progression of CLL on treatment due to development of RS in 28–58% of patients [18,19,20]. In a report by Maddocks et al. (2015), patients with relapsed CLL who developed RS while on ibrutinib therapy tended to have earlier progression of disease; the estimated cumulative incidence of RS at 12 months was 4.5%, whereas the cumulative incidence of CLL progression without RS at 12 months was 0.3% [20]. Miller et al. (2017) reported that near-tetraploidy in patients with CLL treated with ibrutinib was associated with a higher risk of progression to RS [21]. A study evaluating the use of venetoclax reported 16% of patients developed RS [22]. In these studies, patients were being treated for relapsed or refractory CLL, which makes it difficult to ascertain how often cases of early transformation on novel treatment may be due to underlying undiagnosed RS prior to treatment initiation or presence of high-risk features, which prompted treatment with targeted agents (e.g., del(17p)/ TP53 inactivation). A recent study evaluating patients diagnosed with CLL and RS between 2000 and 2023 analyzed the incidence of RS prior to the utilization of novel agents (i.e., pre-2014) and in the novel agent era (i.e., post-2014 FDA approval of ibrutinib) [16]. There was an increased risk of RS noted in the cohort of patients treated for CLL in the pre-novel agent era compared to the novel agent era, though the difference was not statistically significant. The authors theorized that the difference in trends towards RS development may be due to exposure to chemoimmunotherapy and clonal evolution in the pre-novel agent era or may be related to suppression of RS development with the use of more effective novel agents, though this requires further investigation [16]. Given this trend, novel immunotherapies for CLL that improve disease control may be associated with decreased rates of progression due to RS development.

## 3. Pathogenesis

DLBCL-RS must be differentiated from aggressive CLL for diagnosis, which can be difficult. Aggressive CLL demonstrates an increase in size and proliferative activity of leukemic cells as well as expansion of nodal proliferation centers [23]. In this disease, lymphocytes are small with low proliferative indices and mitotic activity [24]. The diagnosis of DLBCL-RS requires demonstration of large B cells (either >2x normal lymphocyte or nuclear size larger than/equal to macrophage nuclei) in a diffuse growth pattern [23,25]. DLBCL-RS is typically associated with a high Ki67 proliferation index [26,27].

In the majority of cases of DLBCL-RS, large cells are derived from a CLL clone, as demonstrated by IgHV sequencing; however, in approximately 20% of cases, RS is clonally unrelated [28]. Clonal evolution can occur with acquisition of del(17p) or other cytogenetic abnormalities; Rossi et al. (2011) reported that 47% of cases were associated with TP53 aberrations and 26% with cMYC lesions [29]. They also noted that patients with clonally related RS had inferior outcomes compared to clonally unrelated transformation [29]. Unfortunately, determining clonality is not always feasible due to availability of testing or known baseline IgHV status.

Analysis of genomic abnormalities in CLL, DLBCL-RS, and de novo DLBCL demonstrated that cell cycle deregulation via inactivation of TP53 and CDKN2A is a main driver of the transformation from CLL to aggressive lymphoma [30]. In this study, 50% of cases were characterized by TP53 inactivation and/or CDKN2A loss along with MYC activation or 13q loss [30]. A total of 28% of cases were associated with trisomy 12, often with NOTCH1 lesions, and the remaining cases were heterogeneous regarding genetic abnormalities [30]. The investigators noted that the genetic complexity of DLBCL-RS was intermediate between that of CLL and de novo DLBCL, and that the genomic profile of RS was clearly different from that of de novo DLBCL [30]. Fabbri et al. (2013) noted that NOTCH1 mutations were infrequent in de novo DLBCL but were noted in 8.3% of cases of CLL at diagnosis and at much higher rates in refractory CLL (20.8%) and RS (31%). It has been proposed that smaller subclones with these genetic abnormalities may be present for years prior to the diagnosis of transformation, and perhaps clonal selection occurs due to the natural history of the disease or treatment effect [31].

Reactivation of Epstein–Barr virus (EBV) has been identified as a possible trigger for transformation of CLL to DLBCL. In a study analyzing a cohort of 31 patients with RS, 7 (23%) were noted to be EBV-positive; these cases demonstrated CLL unrelated-IgHV sequences more frequently than EBV-negative tumors. Notably, clonally related and unrelated EBV-positive DLBCL were both associated with prior administration of chemoimmunotherapy, suggesting that EBV reactivation during periods of immunosuppression could transform either CLL cells or other non-tumor B cells into DLBCL [32].

The role of programmed cell death 1 (PD-1) and its ligands (PD-L1) in the pathogenesis of RS has also been investigated. PD-L1 may be upregulated by cancer cells to evade anti-tumor immunity. Interestingly, a study published by Behdad et al. (2019) demonstrated that there was increased expression of PD-1 in cases of RS. They did not notice similar PD-1 expression in cases of de novo DLBCL, suggesting that the PD-1/PD-L1 pathway may be utilized more specifically in the case of transformation to inhibit anti-tumor responses [33].

## 4. Clinical Presentation and Diagnosis

Transformation to an aggressive lymphoma should be suspected in patients who develop rapid deterioration in clinical status including the development of “B” symptoms (e.g., fevers/chills, night sweats, weight loss), lymphadenopathy, or symptomatic splenomegaly. Laboratory findings can include rising LDH, and worsening anemia or thrombocytopenia.

If there is clinical suspicion for transformation of CLL, a PET/CT should be obtained. An SUVmax ≥5 in the setting of progressive disease has been associated with an average sensitivity of 87% (range 71–96%), specificity of 49% (4–80%), positive predictive value of 41% (16–53%), and negative predictive value of 84% (33–97%) for the detection of RS [34]. A study of 240 patients with CLL demonstrated that 90% of patients with histologically confirmed RS had a median tumor SUVmax of >10, which was significantly different from patients with aggressive CLL progression [35]. This study noted that SUVmax > 10 was associated with 91% sensitivity and 95% specificity for identification of RS by PET [35]. Additionally, a higher SUVmax on pre-treatment PET has been associated with poorer outcomes (SUV ≥ 10 with a median OS 6 months vs. SUV < 10 with OS 21 months) [36]. Similarly, disease on both sides of the diaphragm was associated with worse OS [36]. Diagnosis should be made with excisional biopsy of the most SUV avid node on PET/CT. It is possible for different cell populations to coexist, so it is vital to obtain tissue to differentiate between aggressive CLL and transformation to DLBCL-RS. A bone marrow biopsy should also be completed. Ideally, samples should be sent to test for clonality of IgHV and for complete mutational analysis.

## 5. Treatment

### 5.1. Chemotherapy and Chemoimmunotherapy

Due to the rarity of RS as well as difficulties in its diagnosis, most evidence guiding treatment has been derived from phase I/II trials or retrospective studies rather than randomized clinical trials. The standard of care for DLBCL-RS has been chemoimmunotherapy (CIT).

A study of 29 patients with RS treated with hyperCVAD (cyclophosphamide, vincristine, doxorubicin, dexamethasone) demonstrated an ORR of 41%. Of those patients achieving a CR (38%), 4 patients relapsed within 12 months of remission. The median OS was 10 months and significantly improved for patients achieving a CR (19 months vs. 3 months) [37]. The addition of rituximab to this regimen did not demonstrate notable improvement in outcomes [38]. In a study of 49 patients (30 with RS and 19 with refractory CLL), patients were treated with rituximab and hyperCVAD with an ORR of 41% (CR 18%) and median OS of 8 mo. The PFS and OS rates at 12 months were 27% and 39% [38]. Additionally, treatment with hyperCVAD both with and without rituximab were associated with significant toxicity including hematotoxicity and infectious complications.

Oxaliplatin-based regimens have also been investigated in the setting of RS and refractory CLL. Tsimberidou et al. (2013) reported on the OFAR1 regimen (oxaliplatin, fludarabine, cytarabine, rituximab) with a 50% ORR in the RS cohort [39]. However, the initial OFAR1 regimen was associated with myelosuppression.

R-CHOP (rituximab, cyclophosphamide, doxorubicin, vincristine, prednisone) has an established role in de novo DLBCL and has demonstrated some efficacy in the setting of DLBCL-RS. Langerbeins et al. (2014) conducted a phase II study evaluating R-CHOP for patients with RS, as well as high risk CLL and CLL with autoimmune cytopenias. In the RS cohort, the ORR was 67%, with a median PFS of 10 months and median OS of 21 months. Hematotoxicity was prevalent with this regimen (92% of all patients) and severe infections occurred in 28% of patients, with early discontinuation of therapy in 45% [40]. A phase II study of ofatumumab with CHOP evaluated 37 patients with RS with an ORR of 46% (CR 27%, PR 19%), median PFS of 6.2 months and median OS of 11.4 months [41]. Overall, this regimen was determined to provide minimal benefit compared with R-CHOP. Outcomes with R-EPOCH (rituximab, etoposide, prednisone, vincristine, cyclophosphamide, doxorubicin) were modest, with an ORR of 37% (CR 20%), median PFS 3.5 months and median OS 5.9 months [42]. In this study, complex karyotype and number of prior treatments were associated with worse outcomes [42]. Salvage therapy with platinum and cytarabine regimens (DHAP: dexamethasone, cytarabine, cisplatin; ESHAP: etoposide, methylprednisolone, cytarabine, cisplatin) had a similar ORR of 43% and CR of 25% in patients with RS [43]. With these regimens, there was a median PFS of 6.9 months and OS of 8.3 months [43]. Overall, results of these studies demonstrate that RS is associated with limited response to chemoimmunotherapy, with more intensive chemotherapy regimens causing unacceptable toxicity for limited, if any, clinical benefit compared to conventional anthracycline based chemotherapy approaches.

### 5.2. Hematopoietic Stem Cell Transplantation

Hematopoietic stem cell transplantation (HSCT) has been associated with durable responses in DLBCL-RS and has been incorporated into the treatment paradigm for DLBCL-RS given the modest overall survival associated with CIT. In a 2006 study, Tsimberidou et al. reported on 20 patients who underwent stem cell transplantation (SCT) as either consolidation or salvage therapy after receiving chemotherapy or CIT. They found that patients who underwent allogeneic SCT (alloSCT) after achieving remission had longer survival than those who underwent salvage alloSCT or who received no additional therapy after remission with initial systemic therapy [44]. A retrospective study of 59 patients with RS described outcomes for both alloSCT and autologous SCT (autoSCT) conducted between 1997 and 2007 [41]. In this study, alloSCT and autoSCT were associated with a 3-year PFS of 27% and 45% and an OS of 36% and 59%, respectively. Notably, 82% of patients in the autoSCT cohort were in remission prior to transplant, while this was the case for only 60% of the alloSCT cohort. Reduced intensity conditioning, used in 72% of patients, was associated with superior PFS after alloSCT [45]. Outcomes of an analysis of 66 patients who underwent alloSCT between 2008 and 2018 similarly demonstrated 3-year OS of 39% and PFS of 29% [46]. Patients in CR prior to transplant had significant improvement in PFS (39% vs. 21%). The reported incidence of grade II–IV acute graft versus host disease (GVHD) was 41% at day 100; the incidence of chronic GVHD was 53% at 3 years, suggesting that GVHD may be a limitation to alloSCT in this patient cohort [46]. A larger study evaluated 118 alloSCT and 53 autoSCT patients with 3-year PFS 43% and 48% and OS 52% and 57%, respectively [47]. Outcomes between the cohorts were difficult to compare due to differences in patient characteristics including pre-transplant remission status (66% in autoSCT vs. 34% in alloSCT), prevalence of 17p deletion (7% in autoSCT vs. 33% in alloSCT), and history of treatment with novel agents (10% in autoSCT vs. 39% in alloSCT) [47]. Kim et al. reported on 28 patients with RS who underwent alloSCT, noting a median PFS of 11.2 months and 8 documented relapses (7 with RS and 1 with CLL) [48]. Notably, though consolidation alloSCT is generally recommended, often patients with RS are ineligible for transplant. A 2023 study by Puckrin et al. reported on 99 patients who were diagnosed with DLBCL-RS between 2006 and 2020 in their geographic region; of these patients, only 20% (n = 20) were deemed eligible for transplant and only 25% of these patients successfully underwent alloSCT (n = 5). They determined that reasons for transplant ineligibility included age, treatment-naïve CLL, insufficient medical fitness, poor performance status, and disease progression [49]. Overall, though alloSCT has demonstrated durability of response for DLBCL-RS, it is an option for treatment that is limited by patient eligibility as well as incidence of GVHD.

### 5.3. Small Molecule Targeted Therapy

B-cell receptor signaling is integral to tumor cell survival in CLL and Bruton’s tyrosine kinase is essential in this pathway. Byrd et al. (2013) reported on outcomes of 85 patients with relapsed/refractory CLL who were treated with ibrutinib, a BTK inhibitor (BTKi). In this study, the ORR was 71% with an estimated PFS of 75% and OS of 83% at 26 months; however, despite this response, 7 patients on trial developed RS [50]. Given these results, there was concern that the role of BTKi in RS would be limited. Tsang et al. (2015) initially demonstrated a response to ibrutinib in 4 patients with previously treated RS, with 75% ORR. Unfortunately, responses in this series were short-lived, with a median duration of response of 6.1 months [51]. Monotherapy with acalabrutinib was demonstrated to have an ORR of 38–40% with a median duration of response of 5–6.2 months and median PFS 3–3.2 months [52,53]. The most common adverse effects were diarrhea, headache, anemia, and neutropenia [53]. Zanubrutinib monotherapy in a small cohort of patients with RS (n = 13) had a 61.5% ORR with a median duration of response of 25.4 months [54]. Though an overall promising result, all patients experienced at least one treatment-related adverse effect and 69.2% of patients had at least a grade 3 AE [54].

Pirtobrutinib, a noncovalent BTKi, has been established for use in the setting of relapsed/refractory CLL and mantle cell lymphoma. Wierda et al. (2024) reported on the safety and efficacy of pirtobrutinib in a subgroup of patients with DLBCL-RS enrolled in the phase I/II BRUIN study [55]. Of the 82 patients, 90% had received at least one prior line of therapy for RS and 74% had received a covalent BTKi for CLL or RS. The ORR was 50% w/13% of patients achieving CR and 37% with PR. Neutropenia was the most common grade 3 or worse adverse event (23% of patients) [55]. National guidelines recommend pirtobrutinib monotherapy for patients who are not candidates for chemoimmunotherapy [56]. Pirtobrutinib is currently being studied in combination with venetoclax and obinutuzumab (NCT05536349).

The anti-apoptotic protein BCL2 is overexpressed in malignant cells, and its inhibition has been studied in the setting of DLBCL-RS. In an initial phase I study, venetoclax was demonstrated to have a 43% response; however, the duration of response was short [57]. In combination with DA R-EPOCH, venetoclax was shown to have an ORR of 62% with a 50% CR. With combination therapy, median PFS was 10.1 months and median OS was 19.6 months. Grade 3 neutropenia (65%) and thrombocytopenia (50%) were documented; there were no cases of tumor lysis syndrome with venetoclax administration [58]. Outcomes of venetoclax in combination with a Bruton’s tyrosine kinase inhibitor (n = 28), R-CHOP (n = 13) or other chemoimmunotherapy (n = 21) were reported in a retrospective study by Hampel et al. (2024). The ORR, CR, median PFS, and median OS were 36%/25%/4.9 months/14.3 months with BTKi, 54%/46%/14.9 months/not reached with R-CHOP, and 52%/38%/3.3 months/9 months with other CIT. Infection rates were similar across the different treatment groups; CIT was associated with more frequent grade 3–4 neutropenia and thrombocytopenia. Notably, del(17p) and TP53 mutated CLL were associated with a lower CR rate in the subgroup of patients who were venetoclax-naïve [59]. Overall, these data suggest that the combination of venetoclax with chemoimmunotherapy can lead to durable remission, especially in patients who lack del(17p)/TP53 mutations.

A small report of the use of idelalisib, a PI3Kδ (phosphatidylinositol 3-kinase delta) inhibitor, in RS (n = 4) demonstrated on ORR of 75% with 1 patient achieving CR and 2 with PR; an estimated 6-month OS was 60% [60].

### 5.4. Protein Degrader Therapy

While BTK inhibitors have demonstrated some efficacy in both CLL and RS, these diseases can progress while on BTKi due to development of mutations conferring resistance. As such, BTK degraders were generated, which attempt to bypass resistance mutations by binding BTK and E3 ligase to result in BTK degradation via ubiquitination. BGB-16673, one such small molecule, was studied in 26 patients with relapsed/refractory CLL (n = 10), RS (n = 1), as well as other B cell malignancies in a phase I trial. Of 18 evaluable patients in the total study population, 67% demonstrated response (including 83% of patients with CLL) [61]. The FDA provided fast track approval in 2024 for use of BGB-16673 for patients with relapsed/refractory CLL who had previously been treated with at least two lines of therapy [62]. NX-5948 is another BTK degrader that has been evaluated in patients with relapsed/refractory CLL; of 49 evaluable patients, there was an ORR of 75.5% at week 8 and an increase to 84.2% by week 16 [63]. These new targeted agents have had promising results in CLL thus far and require further investigation for use in the treatment of DLBCL-RS.

### 5.5. Checkpoint Inhibitor Therapy

It has been proposed that upregulation of PD-1/PD-L1 allows for immune evasion in RS. Several studies have been conducted to evaluate the use of immune check point inhibitors (ICI) as monotherapy as well as in combination with small molecule targeted agents. Monotherapy with pembrolizumab has shown conflicting results in the treatment of DLBCL-RS. An initial phase 2 study included 9 patients with RS, with an ORR of 44%, median PFS of 5.4 months, and median OS of 10.7 months [64]. However, KEYNOTE-170 utilized pembrolizumab for 23 patients with RS (21 with DLBCL and 2 with HL), with an ORR of 13% and only 1 PR in the DLBCL cohort [65]. In both studies, patients had received prior therapy for CLL/RS.

There have been several reports on the use of ICI with BTK inhibitors. Younes et al. (2019) reported on 20 patients with RS who were treated with nivolumab and ibrutinib with a 65% overall response rate [66]. In a study of 24 patients treated with the same regimen, 42% of patients responded with a median duration of response of 15 months. The median OS was greater for patients with a response (25 months) compared to non-responders (7.6 months). Notably, the majority of responses were noted in patients who were BTKi-naïve [67]. A phase I/II study of 7 patients with relapsed RS who were treated with tislelizumab and zanubrutinib showed a 42.9% ORR; however, authors of this study noted the limitations of their sample size in determining the efficacy of this regimen [54]. A larger phase II trial of tislelizumab and zanubrutinib for RS included 48 patients with 58.3% ORR (18.8% CR, 39.6% PR) and a median PFS 10 months [68]. Of note, only 20.8% of patients in this study had received prior treatment for RS, and most patients who developed progression of disease subsequently were treated with CIT (including 8 patients who proceeded to alloSCT), indicating that salvage therapies remained feasible for these patients [68].

There are ongoing studies evaluating the combination of BCL2i or PI3K inhibitors with immune checkpoint inhibitors and additional agents. A phase I/II trial evaluated the combination of venetoclax with umbralisib and ublituximab with 40% ORR, though in a smaller cohort of 5 patients [69]. The combination of venetoclax, atezolizumab, and obinutuzumab in venetoclax-naïve patients with untreated or relapsed/refractory RS demonstrated an ORR of 100% (n = 7, CR 71%, PR 29%) [70]. The MOLTO phase II clinical trial evaluated the same combination of venetoclax, atezolizumab, and obinutuzumab in 28 patients with untreated RS, with an ORR of 67.9%. Grade 3 or worse adverse events were reported in 61% of patients, and two deaths were attributed to adverse events while on study (sepsis and fungal pneumonia) [71].

The combination of pembrolizumab with umbralisib and ublituximab demonstrated an ORR of 37.5% (25% CR, 12.5% PR) in 8 patients [72]. Umbralisib has since been withdrawn from market by the FDA due to safety concerns. A phase I study including 7 patients treated with a regimen of obinutuzumab, high-dose methylprednisolone, and lenalidomide for RS demonstrated an ORR of 43%. All three patients who achieved a response subsequently underwent HSCT with durable remission, suggesting that this regimen may be appropriate for bridging to transplant [73]. Given evidence of improved efficacy with combination therapy, additional studies evaluating the use of ICI with small molecule targeted agents are warranted.

### 5.6. Bispecific Antibodies/Bispecific T Cell Engager Therapy

Treatment of hematologic malignancies with immunotherapy has expanded to the use of bispecific antibodies (BsAbs), which recruit T cells to tumor cells. A phase I study of glofitamab, a CD20xCD3 BsAb, included 10 patients with RS; of these, 6 patients were assessed, with an 83% ORR (50% CR, 33% PR) [74]. Blinatumomab, a CD19xCD3 BsAb, has been utilized in several phase II trials. Blinatumomab monotherapy was evaluated in a study including 9 patients with RS who had received prior treatment for CLL and/or RS; median OS was 10.3 months, median PFS 1.9 months, and ORR 22%. Reversible neurotoxicity was the most common adverse effect, followed by cytokine release syndrome [75]. The BLINART trial treated therapy-naïve patients with R-CHOP for 2 initial cycles and then allocated those with poor response to chemoimmunotherapy to blinatumomab induction. Of 39 patients who started systemic therapy, 25 were treated with blinatumomab with CR in 20% of patients after induction and PR in 16%. The ORR was 46%, which was calculated based on the total strategy (including patients who did not require BsAb induction). The most common adverse effects were fevers (36%), hematotoxicity (anemia 24%, lymphopenia 24%), neurotoxicity (20%), and cytokine release syndrome (CRS) (16%) [76]. Epcoritamab, a CD20xCD3 BsAb, was evaluated in a study including 35 patients with RS; the ORR in 26 evaluable patients was 50% with a 35% CR rate. At 9 months follow up, 64% of those with a response and 89% of those with CR were progression free [77]. These studies demonstrate the role of bispecific antibodies in the treatment of DLBCL-RS, and additional studies are required to improve efficacy with this therapy.

### 5.7. Chimeric Antigen T Cell Therapy

The use of chimeric antigen receptor T cell therapy (CAR-T) directed against CD19 has been studied for DLBCL-RS. An early study published by Kittai et al. (2020) evaluated the use of axicabtagene ciloleucel in 8 patients with DLBCL-RS; there was an ORR of 100% with 62.5% of patients achieving CR. Treatment was complicated by development of cytokine release syndrome (CRS) in all patients, as well as grade ≥3 immune effector cell-associated neurotoxicity syndrome (ICANS) in 3 patients [78]. Winter et al. (2024) reported on a study of 30 patients with RS who were treated with lisocabtagene maraleucel. At a median follow-up of 12.3 months, the ORR was 76% (n = 29) and CR was 66%. The 12-month duration of response was 77%, with an estimated 12-month PFS of 54% and OS of 67%. CRS and ICANS occurred in 70% (grade 3–5 6%) and 47% (grade 3–5 27%) of patients, respectively [79].

An international retrospective study of 69 patients treated with CAR-T (axicabtagene ciloleucel in 64% of patients, tisagenlecleucel in 25%, lisocabtagene maraleucel in 10%, and brexucabtagene autoleucel in 1%) demonstrated an ORR of 63%, with 46% of patients achieving a CR (median duration of response 27.6 months) [80]. The median PFS was 4.7 months, and the median OS was 8.5 months. Of note, this cohort of patients had been heavily pre-treated for CLL and/or RS with a median of 4 lines of prior therapy, including history of exposure to small molecule targeted agents (BTKi, BCL2i) in 84% of patients [80]. A separate multicenter retrospective study of 30 patients w/DLBCL-RS treated with CD19-directed CAR-T reported a median OS of 9.9 months with an ORR of 57% and CR of 47% at the 100-day mark [81]. Outcomes were compared against those of patients with aggressive B cell lymphoma and transformed indolent non-Hodgkin lymphoma (iNHL) treated with CAR-T, with inferior survival (12-month OS was 45% in patients with RT compared with 62% in de novo DLBCL and 75% in transformed iNHL). Patients were heavily pre-treated with 67% receiving prior therapy directed against CLL and 89% with prior exposure to BTKi or BCL2i [81]. There is interest in combining CAR-T therapy with small molecule targeted agents and/or immune checkpoint inhibition for increased efficacy.

A single patient demonstrated CR in a phase I/II trial of anti-CD19 CAR-NK cell therapy for RS (n = 2 for RS, total n = 11) [82].

### 5.8. Potential New Therapeutic Agents

CDKN2A loss and NOTCH1 mutations have been implicated in the pathogenesis of DLBCL-RS, representing possible new opportunities for targeted treatment. The CDKN2A gene generates transcripts encoding p16(INK4) and p14(ARF) proteins, which are tumor suppressors; loss of this gene allows for cell proliferation. A study by Chakraborty et al. (2021) demonstrated that inactivating lesions of CDKN2A/B and TP53 often occur concurrently in RS, and these tumor cells demonstrate sensitivity to combination therapy with BCR and CDK 4/6 inhibition in vitro and in vivo in murine models [83]. Pan-CDK inhibitors have been studied in relapsed/refractory CLL and have demonstrated some promise. Byrd et al. (2007) reported on the use of flavopiridol (alvocidib) in 42 patients with CLL, with 45% ORR and a median duration of response exceeding 12 months [84]. A phase I dose escalation study of PRT2527, a CDK9 inhibitor, in relapsed/refractory hematologic malignancies including RS is currently active [85]. Further investigations are warranted into the use of novel CDK inhibitors or combination therapy in RS.

Notch1 also plays a role in the regulation of stem/progenitor cells and mutations have been seen in various hematologic malignancies. There have been several attempts to inhibit Notch1 signaling, including the development of monoclonal antibodies, γ-secretase inhibitors, and Notch transcription complex inhibitors [86]. Casulo et al. (2016) reported on the use of a novel Notch1 targeting antibody, brontictuzumab, in patients with known N1 activating mutations and hematologic malignancies, including patients with CLL (n = 5) and DLBCL (n = 6). In this phase I dose escalation trial, brontictuzumab was felt to be safe, though further studies are necessary to evaluate efficacy [87]. Other studies have utilized Notch1 inhibitors for solid tumors. For example, LY3039478, an inhibitor which blocks the proteolytic activity of γ-secretase resulting in reduced Notch signaling, has been studied in heavily pre-treated patients with advanced or metastatic disease and demonstrated clinical activity in breast cancer, leiomyosarcoma, and adenoid cystic carcinoma [88]. Carrillo-Tornel et al. (2021) evaluated bone marrow and blood samples from CLL patients and found that 9.8% of patients had Notch1 mutations, which were associated with a higher percentage of cells in the S phase compared with Notch wild-type. They subsequently exposed these cells to palbociclib, a CDK 4/6 inhibitor, and noted a marked decrease in cells in the S phase (16% to 0.64%), which suggests that there may be additional opportunities for targeted therapies [89].

## 6. Future Directions

**Figure 1:**
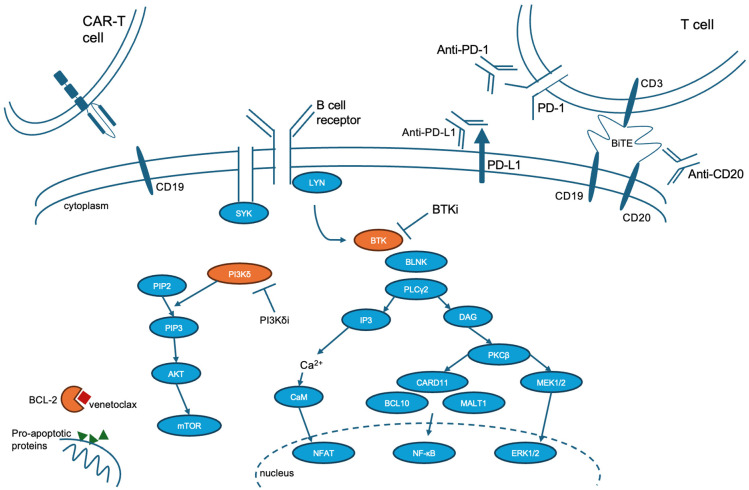
Mechanism of action of treatments for DLBCL-RS. CAR-T = chimeric antigen receptor T cell, PD-1 = programmed cell death protein 1, PD-L1 = programmed cell death protein ligand 1, BiTE = bispecific T cell engager, BTKi = Bruton’s tyrosine kinase inhibitor, PI3Kδi = phosphatidylinositol 3-kinase delta inhibitor, BCL-2 = B cell lymphoma 2.

Of the currently studied treatment options for DLBCL-RS (Figure 1), zanubrutinib and BCL2i-CIT combination therapies have demonstrated the greatest efficacy in terms of median OS (Table 2). In an effort to identify additional treatment options for DLBCL-RS, there are numerous trials that are currently ongoing [85]. Several trials are aimed at identifying whether BsAbs, as monotherapy or in combination regimens, will be efficacious in RS (e.g., NCT04623541 for epcoritamab, NCT06043674/NCT06186648 for glofitamab, NCT06521996 for mosunetuzumab). Other studies are evaluating the safety and efficacy of BTKi in combination with ICI and BCL2i (e.g., NCT04271956, NCT05388006), or with CIT (e.g., NCT03899337). Pirtobrutinib is being evaluated in combination with BCL2i and anti-CD20 monoclonal antibody (mAb; NCT05536349). The combination of BCL2i with ICI and anti-CD20 mAb is also being studied in relapsed/refractory RS (NCT02846623). Additional studies are evaluating the use of CD19-directed CAR-T (lisocabtagene maraleucel) in combination with BTKi +/− ICI (NCT05873712, NCT05672173). Antibody drug conjugates (ADC) have not yet been established for use in DLBCL-RS, with ongoing trials of polatuzumab vedotin, a CD79b-directed ADC that has shown efficacy in de novo DLBCL (NCT04679012), and zilovertamab vedotin (ZV), which targets ROR1 on the surface of RS cells (NCT05458297). A phase I trial of ZV demonstrated objective tumor response in 60% of patients with DLBCL (n = 5); however, only 1 patient with RS was included and did not demonstrate a response [90]. The planned phase II trial will evaluate ZV both as monotherapy and in combination therapy.

## 7. Conclusions

Richter’s transformation of CLL to DLBCL is a distinct disease process that is associated with an overall poor prognosis, particularly for clonally related transformation. Some of the genetic aberrations associated with RS include lesions of TP53, cMYC, CDKN2A, and NOTCH1; additionally, PD-1 expression has been associated with DLBCL-RS. The standard of care therapy for DLBCL-RS is chemoimmunotherapy; however, responses to CIT alone are limited with PFS up to 10 months and significant risk of hematotoxicity and infection. Given the frequency of del(17p) or inactivation of TP53 in DLBCL-RS, this may contribute to poorer responses to CIT. Consolidative HSCT has demonstrated durability of response but there are barriers to transplants that limit its use in RS, including age, performance status, and disease progression. In the last decade, investigations into the use of BTKi, BCL2i, ICI, and BsAb monotherapies have shown some efficacy, though without improved survival outcomes. Notably, the combination of BCL2i with CIT has demonstrated promising results, with up to 62% overall response rate. Numerous trials are ongoing to identify therapies with improved efficacy and durability of response for DLBCL-RS. Increased focus has been placed on combination therapies given demonstration of improved response rates when utilizing different mechanisms of action to target RS (e.g., BTKi with CIT, BTKi with ICI, and BCL2i, CAR-T with BTKi, etc.). Additionally, antibody drug conjugates (ADC) have not yet been established for use in DLBCL-RS, with ongoing trials of polatuzumab vedotin and zilovertamab vedotin. Given its overall poor response to therapy, treatment of DLBCL-RS continues to pose a challenge to clinicians; further investigation into combined regimens will hopefully provide insight into optimal treatment of this disease.

## Figures and Tables

**Table 1:** Rates of Richter’s syndrome development. NR = not reported.

Patients with CLL (n)	Rate of Development Richter’s Syndrome	Median Time to Onset/ Cumulative Incidence Rate from Diagnosis	Study Dates	Reference
1641	37/1641 (2.3%)	1 year 0.5%	2000–2011	Parikh 2013 [3]
84	4/84 (4.8%)	NR	2011–2017	Ahn 2018 [12]
2975	103/2975 (3.5%)	NR	1999–2016	Al-Sawaf 2021 [5]
533	23/533 (4.3%) total study population 10/268 (3.7%) in acalabrutinib arm 13/265 (4.9%) in ibrutinib arm	7.1 months in acalabrutinib arm/11.5 months in ibrutinib arm	2015–2020	Byrd 2021 [13]
675	28/675 (4.2%) total study population 26/563 (4.6%) in ibrutinib arm 2/112 (1.8%) in idelalisib+ rituximab arm	NR	-	Morabito 2021 [14]
382	13/382 (3.4%)	NR	2014–2020	Seymour 2022 [15]
3347	82/3347 (2.4%)	1 year 0.6%, 5 year 1.8%, 10 year 3.0%	2000–2023	Hampel 2023 [16]

**Table 2:** Treatments for DLBCL-RS. ORR = overall response rate, CR = complete response, PFS = progression free survival, OS = overall survival, NR = not reported, BTKi = Bruton’s tyrosine kinase inhibitor, CAR-T = chimeric antigen receptor T cell therapy, NHL = non-Hodgkin lymphoma.

Treatment	Patients (n)	ORR/CRR (%)	Median PFS (months)	Median OS (months)	Reference
Ibrutinib	4	75%/25%	6.1	NR	Tsang 2015 [51]
Acalabrutinib	25 29	40%/8% 38%/14%	3.2	NR	Eyre 2021 [53] Hillmen 2016 [52]
Zanubrutinib	13	61.5%/15.4%	17.3	29.3	Tam 2023 [54]
Pirtobrutinib	82	50%/13%	3.7	12.5	Wierda 2024 [55]
Venetoclax	7	43%/0%	NR	NR	Davids 2017 [57]
Venetoclax + DA R-EPOCH	26	62%/50%	10.1	19.6	Davids 2022 [58]
Venetoclax + BTKi	28	36%/25%	4.9	14.3	Hampel 2024 [59]
Venetoclax + R-CHOP	13	54%/46%	14.9	Not reached	Hempel 2024 [59]
Idelalisib	4	75%/25%	NR	NR	Visentin 2019 [60]
Pembrolizumab	9 23	44%/11% 13%/0%	5.4 1.6	10.7 3.8	Ding 2017 [64] Armand 2020 [65]
Nivolumab + ibrutinib	20 24	65%/10% 42%/33%	5.0 NR	10.3 13	Younes 2019 [66] Jain 2023 [67]
Tislelizumab + zanubrutinib	48	58.3%/18.8%	10	NR	Al-Sawaf 2023 [68]
Umbralisib + ublituximab + venetoclax	5	40%/40%	NR	NR	Hill 2024 [69]
Atezolizumab + obinutuzumab + venetoclax	7 28	100%/71% 67.9%/28.6%	NR NR	NR NR	Jain 2021 [70] Tedeschi 2024 [71]
Pembrolizumab + umbralisib + ublituximab	8	37.5%/25%	NR	NR	Mato 2018 [72]
Obinutuzumab + high-dose methylprednisolone + lenalidomide	7	43%/29%	5	17	Heyman 2022 [73]
Glofitamab	10	83%/50%	2.9 (all aggressive NHL)	NR	Hutchings 2021 [74]
Blinatumomab	9 25	22%/11% 46%/20%	1.9 3.8	10.3 9.1	Thompson 2022 [75] Guieze 2024 [76]
Epcoritamab	35	50%/35%	NR	NR	Kater 2024 [77]
Axicabtagene ciloleucel	8	100%/62.5%	NR	NR	Kittai 2020 [78]
Lisocabtagene maraleucel	30	76%/66%	NR	NR	Winter 2024 [79]
CD19 CAR-T cell	69	63%/46%	4.7	8.5	Kittai 2024 [80]

## Data Availability

No new data were collected.

## Abbreviations

The following abbreviations are used in this manuscript:

RS	Richter’s syndrome
DLBCL	Diffuse large B cell lymphoma
CLL	Chronic lymphocytic leukemia
CIT	Chemoimmunotherapy
HSCT	Hematopoietic stem cell transplantation
ICI	Immune checkpoint inhibitor
CAR-T	Chimeric antigen receptor T cell
BsAb	Bispecific antibody
